# Intracerebral infection with dengue-3 virus induces meningoencephalitis and behavioral changes that precede lethality in mice

**DOI:** 10.1186/1742-2094-8-23

**Published:** 2011-03-09

**Authors:** Debora CG Amaral, Milene A Rachid, Marcia C Vilela, Roberta DL Campos, Gustavo P Ferreira, David H Rodrigues, Norinne Lacerda-Queiroz, Aline S Miranda, Vivian V Costa, Marco A Campos, Erna G Kroon, Mauro M Teixeira, Antonio L Teixeira

**Affiliations:** 1 Laboratório de Imunofarmacologia, Departamento de Bioquímica e Imunologia, Instituto de Ciências Biológicas (ICB), UFMG, Belo Horizonte, Brazil; 2 Laboratório de Vírus, Departamento de Microbiologia, Instituto de Ciências Biológicas (ICB), UFMG, Belo Horizonte, Brazil; 3 Centro de Pesquisas René Rachou, Belo Horizonte, Brazil

## Abstract

**Background:**

Dengue, one of the most important arboviral diseases of humans, may cause severe systemic disease. Although dengue virus (DENV) has been considered to be a non-neurotropic virus, dengue infection has been associated recently with a series of neurological syndromes, including encephalitis. In this work, we evaluated behavioral changes and inflammatory parameters in C57BL/6 mice infected with non-adapted dengue virus 3 (DENV-3) genotype I.

**Methods:**

C57BL/6 mice received 4 × 10^3 ^PFU of DENV-3 by an intracranial route. We evaluated the trafficking of leukocytes in brain microvasculature using intravital microscopy, and evaluated chemokine and cytokine profiling by an ELISA test at 3 and 6 days post infection (p.i.). Furthermore, we determined myeloperoxidase activity and immune cell populations, and also performed histopathological analysis and immunostaining for the virus in brain tissue.

**Results:**

All animals developed signs of encephalitis and died by day 8 p.i. Motor behavior and muscle tone and strength parameters declined at day 7 p.i. We observed increased leukocyte rolling and adhesion in brain microvasculature of infected mice at days 3 and 6 p.i. The infection was followed by significant increases in IFN-γ, TNF-α, CCL2, CCL5, CXCL1, and CXCL2. Histological analysis showed evidence of meningoencephalitis and reactive gliosis. Increased numbers of neutrophils, CD4^+ ^and CD8^+ ^T cells were detected in brain of infected animals, notably at day 6 p.i. Cells immunoreactive for anti-NS-3 were visualized throughout the brain.

**Conclusion:**

Intracerebral infection with non-adapted DENV-3 induces encephalitis and behavioral changes that precede lethality in mice.

## Background

Dengue, one of the most important arboviral human diseases, is a serious cause of morbidity and mortality in tropical and subtropical regions of the world. Approximately 2.5 billion people are at risk of being infected by dengue virus [1]. The dengue virus (DENV) comprises four serotypes - DENV-1, DENV-2, DENV-3, and DENV-4, all of which appear to be present in 22 of the 27 states of Brazil [2-4].

Although DENV has been considered a non-neurotropic virus in humans, some authors have described the presence of the virus in cerebrospinal fluid (CSF), and dengue antigens in brain tissue [5,6]. Moreover, infection has been associated with encephalitis, acute disseminated encephalomyelitis, neuropathies, and Guillain-Barré syndrome [6-8]. Therefore, dengue infection should be considered as a possible cause of encephalitis in endemic regions [6]. Patients with dengue encephalitis can present headache, neck stiffness, intermittent tremors, altered consciousness, abnormal coordination, convulsions, and coma [5,6].

Viral, host, and environmental factors contribute to the pathogenesis and progression of the disease. The use of animal models has been relevant to mimic viral encephalitis and to provide mechanisms for comprehension of its pathogenesis [9]. To study the immune response elicited specifically in the CNS compartment and to prevent the spurious influence of the peripheral immune system, it is necessary to use an intracranial route, i.e. to inoculate the virus directly in the brain. We have previously evaluated the immune response in a model of severe Herpes simplex virus type 1 (HSV-1) encephalitis determined by intracerebral inoculation of the virus [10,11]. Interestingly enough, we have demonstrated a differential role for TNFR1 in HSV-1 infection depending on the route of viral inoculation. While TNFR1 seems to play a relevant role in control of viral replication in the CNS when HSV-1 is inoculated by the intracranial route, TNF-α seems to protect against encephalitis by a mechanism independent of TNFR1 when HSV-1 is inoculated in the periphery [12]. Metalloproteinases (MMP) have also been involved in the development of HSV-1 encephalitis by causing damage to the cerebral vasculature and, hence, favoring transmigration of leukocytes and CNS damage [13].

In the present work, we aimed to study behavioral symptoms, leukocyte traffic, and inflammatory parameters in the brain of C57BL/6 mice infected with a non-adapted human DENV-3 genotype I.

## Methods

### Virus

Viral isolation was performed as described elsewhere [2]. Briefly, 50 μL of a serum sample from a single human patient was incubated with C6/36 cells, and at least three successive passages were conducted for each sample. Microscopic examination of cells inoculated with patient serum showed a clearly visible cytopathic effect with changes in the monolayer such as syncytial cell formation and cytoplasmic vacuoles after the third passage. Supernatants of infected C6/36 cells that showed a typical cytopathic effect were centrifuged at 1680 × *g *for 15 min at 4°C. The supernatants were collected, divided in 0.3-mL aliquots and stored at -70°C until use.

### Mice and animal care

Male C57BL/6 mice, ages 6-9 weeks, were obtained from the Animal Care Facilities of the Institute of Biological Sciences, Federal University of Minas Gerais (ICB-UFMG), Belo Horizonte, Brazil. The Animal Ethics Committee of UFMG approved all experimental procedures used in the present study.

### Infection with non-adapted DENV-3

For DENV infection, mice were handled and kept in a biosafety level 2 (BSL-2) facility. To induce encephalitis, anesthetized C57BL/6 mice were inoculated intracranially with 4 × 10^3 ^plaque-forming units (PFU) of the purified DENV-3 genotype I resuspended in 20 μL of phosphate-buffered saline (PBS). Sham animals were injected intracranially with 20 μL of PBS only. The capacity of this inoculum to induce more evident neurological signs and meningoencephalitis than lower doses has been evaluated previously [14]. After infection, the clinical signs and mortality were observed daily.

### Quantification of virus in brain of DENV-3 infected mice

	Viral quantification in infected mice was performed as previously described [14]. Briefly, the brains were harvested, weighed, homogenized, and microcentrifuged (10 min, 4°C, 6000 × g) to pellet the cell debris. LLC-MK2 cells were cultured overnight in 24-well plates before the media were removed and 0.5 mL of serial sample dilutions (10-fold) were added to individual wells. Plates were incubated for 1 h before media were aspirated and replaced with 0.5 mL of 0.8% methyl-cellulose medium (with 2% FBS). Plates were then incubated for 7 days before the media were removed and the cells were fixed in 4% formaldehyde for 20 min, rinsed in water, stained with crystal violet for 20 min, and rinsed again. Plaques were counted visually, and the concentrations of plaque-forming units per mL (PFU/mL) were calculated.

### SHIRPA screen

	Behavioral and functional parameters were evaluated using a screening battery called SmithKline/Harwell/Imperial College/Royal Hospital/Phenotype Assessment (SHIRPA) [15,16]. After a period of mouse adaptation, the procedure was carried out from day 2 until day 7. For the purpose of analysis, the individual parameters assessed by SHIRPA were grouped into five functional categories (neuropsychiatric state, motor behavior, autonomic function, muscle tone and strength, and reflex and sensory function) [15,16].

### Intravital microscopy

Intravital microscopy of the mouse brain microvasculature was carried out as routinely performed in our laboratory [10]. Briefly, mice were anesthetized by intraperitoneal injection of a mixture of ketamine (150 mg/Kg) and xylazine (10 mg/Kg), and the tail vein was cannulated for administration of fluorescent dyes. A craniotomy was performed using a high-speed drill and the dura mater was removed to expose the underlying pial vasculature. Throughout the experiment, mice were maintained at 37°C with a heating pad and the exposed brain was continuously superfused with an artificial cerebrospinal fluid buffer with ionic mmol/L composition of NaCl 132, KCl 1.95, CaCl_2 _1.71, MgCl_2 _0.64, NaHCO_3 _24.6, dextrose 3.71, and urea 6.7, pH 7.4, at 37°C.

Leukocytes were fluorescently labeled by intravenous administration of Rhodamine 6G- Sigma (0.5 mg/kg body weight) and were observed using a microscope (Olympus B201, x20 objective lens, corresponding to 100 μm of area) outfitted with a fluorescent light source (epi-illumination at 510-560 nm, using a 590-nm emission filter). The number of rolling and adherent leukocytes was determined offline during video playback analysis. Leukocytes were considered adherent to the venular endothelium if they remained stationary for a minimum of 30 s. Rolling leukocytes were defined as white cells moving at a velocity lower than that of erythrocytes within a given vessel. Pial vessels with diameters ranging from 50 to 120 μm were used, as most adhesion occurred in vessels in this size range. Leukocyte adhesion was expressed as the number of cells/100 μm.

### Histopathology and immunohistochemistry

Brains from control and infected mice were collected at days 3 and 6 p.i. and preserved in 10% buffered formalin. Sections of 5 μm thickness were cut at intervals of 10 μm and mounted for hematoxylin and eosin staining. For immunohistochemistry, sections were treated with 3% H_2_O_2 _diluted in Tris-buffered saline (TBS) (pH 7.4) for 30 minutes. For antigen retrieval, tissue sections were immersed in citrate buffer (pH 6.0) for 20 minutes at 95°C. For detection of DENV-3-infected cells an anti-NS3 MAb E1D8 was used in a dilution of 1:200; at 4°C overnight. After incubation, tissue sections were washed with TBS and treated with a labeled streptavidin-biotin kit EnVision^® ^+ Dual Link System-HRP (Dako). Sections were then rinsed in PBS with 3,3'-diaminobenzidine tetrahydrochloride (K3468, Dako) for 5 minutes and stained with Mayer's hematoxylin. Negative controls were obtained by the omission of primary antibodies, which were substituted by 1% PBS-TBS.

Histopathological analysis and scoring were performed in cerebral cortex, cerebellum, hippocampus and brainstem. Each area of the brain was graded on a 0 to 4-point scale: 0 = no pathology; 1 = minimal tissue destruction and/or mild inflammation/gliosis; 2 = mild tissue destruction and/or moderate inflammation/gliosis; 3 = definite tissue destruction (neuronal loss, parenchymal damage) and intense inflammation; 4 = necrosis (complete loss of all tissue elements with associated cellular debris. Meningeal inflammation was assessed and graded as follows: 0 = no inflammation; 1 = one cell layer of inflammation; 2 = two cell layers of inflammation; 3 = three cell layers of inflammation; 4 = four or more cell layers of inflammation [10,17]. The density of necrotic or apoptotic neurons was assessed as not detectable (no cell damage: 0), minimal (<10% of all cells: 1), moderate (10-30% of all cells: 2), and numerous (>30% of all cells: 3) [18].

### ELISA of proteins in cerebral tissue

Brain tissue extracts were obtained from control and infected mice and stored at -20°C. Thereafter, the brain tissue was homogenized in an extraction solution (100 mg of tissue per 1 mL of extraction solution) containing 0.4 mol/L NaCl, 0.05% Tween 20, 0.5% BSA, 0.1 mmol/L phenylmethyl sulfonil fluoride, 0.1 mmol/L benzethonium chloride, 10 mmol/L EDTA, and 20 KI aprotinin, using Ultra-Turrax (Fisher Scientific, Pittsburgh, PA). The brain homogenate was centrifuged at 3000 × *g *for 10 min at 4°C, and the supernatant was collected and stored at -20°C. Concentrations of the cytokines IFN-γ and TNF-α, and of the chemokines CCL2, CCL5, CXCL1, and CXCL2 were determined using ELISA. The brain tissue supernatants were assayed in an ELISA setup using commercially available antibodies, according to the manufacturer's procedures (R&D Systems, Minneapolis, MN).

### Tissue extraction and determination of myeloperoxidase (MPO) activity

Tissue extraction and determination of MPO activity were performed. After processing the tissues, the pellet was weighed, homogenized in 2.5 mL of buffered saline EDTA-sodium phosphate-HCl, pH 4.7 (0.1 M NaCl, 0.02 M Na_3_PO_4_, 0.015 M Na_2_EDTA), and centrifuged at 3000 × *g *at 4°C for 15 min. The pellets were then resuspended in 2.5 mL of 0.05 M sodium phosphate buffer pH 5.4 containing 0.5% hexa-1,6-bis-decyltrimethylammonium bromide (HTAB, Sigma). The suspensions were freeze-thawed three times using liquid nitrogen and finally centrifuged at 3000 × *g *at 4°C for 15 min. MPO activity in the resulting supernatant was assayed by mixing 25 μL of 3,3',5,5'-tetramethylbenzidine (TMB, Sigma) prepared in dimethyl sulfoxide (DMSO, Merck) at a final concentration of 1.6 mM with 100 μL of 0.003% (*v*/*v*) H_2_O_2_, dissolved in 0.05 M sodium phosphate buffer pH 5.4 and 25 μL of the supernatants from tissue sample processing. The assay was carried out in a 96-well microplate and was started by incubating the supernatant sample and the TMB solution (25 μL) for 5 min at 37 °C. Then, H_2_O_2 _was added and the incubation continued for another 5 min. The reaction was terminated by adding 100 μL 4 M H_2_SO_4 _and was quantified colorimetrically at 450 nm in a spectrophotometer (E max-Molecular Devices). Results are expressed as change in OD per gram of wet tissue (implant).

### Flow cytometry and cell sorting

Mice were infected intracranially with 4 × 10^3 ^PFU of DENV-3 as described. On days 3 and 6 post infection, mice were anesthetized and perfused intracardially with PBS to remove both circulating and non-adherent RBCs and leukocytes from the brain. Brains were removed and adherent leukocytes isolated using a previously described protocol with minor modifications [19]. Each sample (n) corresponds to a pool of 2-3 mice brains. Briefly, the brains were collected and homogenized gently using a sterile glass tissue grinder in RPMI 1640 medium containing 5% FCS. Homogenates were passed through a nylon cell strainer (70 μm; Becton Dickinson and Company, Brazil) and cells centrifuged at 400 × g for 10 minutes. The pellet was resuspended on a 35% Percoll gradient (Sigma-Aldrich) and this deposited gently on a 70% Percoll gradient. After centrifugation (1100 × g), the leukocytes were collected at the boundary layer, resuspended in FACS buffer (PBS containing 1% FCS and 0.01% NaN3) and counted. Brain-sequestered cells were stained for extracellular molecular expression patterns using monoclonal antibodies (mAb) against mouse CD3e conjugated to phycoerythrin (PE) (BD Pharmingen San Diego, CA; clone 17A2), CD4 to fluorescein isothiocyante (FITC) (BD Pharmingen San Diego, CA; clone L3T4), CD8α conjugated to Peridinin Chlorophyll Protein Complex (PerCP) (BioLegend; clone 53-6.7) for Mix 1, or Ly-6G conjugated to fluorescein isothiocyante (FITC) (eBioscience; clone RB6-8C5), CD11b conjugated to PE-Cy5 (BioLegend; clone M1/70) for Mix 2 and isotype controls (all from BD Pharmingen San Diego, CA). For each sample, 20000 cells from the lymphocyte population were scored. The frequency of positive cells was analyzed using a gate that included lymphocytes and granulocytes. Limits for the quadrant markers were always set based on negative populations and isotype controls. Cells were acquired on a FACS Calibur flow cytometer (BD Biosciences) and analyzed using FlowJo 7.5.3 software (TreeStar Inc.). Analysis in FlowJo software took into account size (forward light scatter) and granularity (side light scatter) of populations. Frequency in number of an analyzed population in front of total acquired events was used in the construction of graphs.

### Statistical analysis

Data are shown as mean ± SEM. The Kaplan-Meier test was employed to compare survival rates. One-way ANOVA with Tukey's correction was used for multiple comparisons. Statistical significance was set at p < 0.05.

## Results

### Effects of non-adapted human DENV-3 on disease symptoms and mouse survival following intracranial inoculation

**Figure 1:**
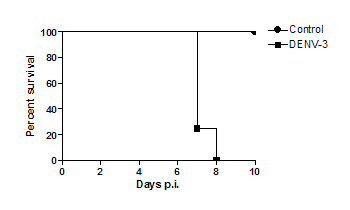
**Time course of survival of mice infected with 4 × 10^3 ^PFU of DENV-3 by the intracranial route (n = 20)**. Control animals (n = 20) received 20 μL of phosphate-buffered saline (PBS).

Following intracranial infection with DENV-3 genotype I, animals presented neurological symptoms of apathy, stereotyped behavior, and seizures. All animals died by day 8 p.i. (Figure 1).

**Figure 2:**
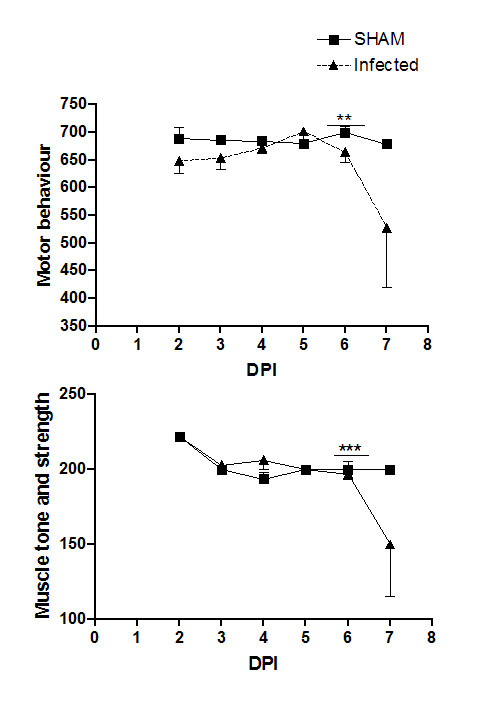
**Visualization of performance (SHIRPA test) of control animals (n = 8) and mice infected with 4 × 10^3 ^PFU of DENV-3 by the intracranial route (n = 8), from days 2 to 7 of infection**. Motor behavior (A) and muscle tone and strength parameters (B) showed significant alterations in the infected group at day 7 p.i. ***p < 0.001 and **p < 0.01.

To better analyze the neurological signs, we performed the SHIRPA battery of behavioral tests in the animals. SHIRPA analysis confirmed that infected mice developed behavioral changes during the course of the disease and that changes occurred predominantly immediately prior to death (Figure 2). Motor behavior (p < 0.01) and muscle tone and strength parameters (p < 0.001) were altered from day 6 onwards and drastically declined on day 7 p.i. (Figure 2 A, B).

### Quantification of virus in brain of DENV-3 infected mice

	Infectious viral titers in mouse brain were determined 3, 5, and 6 days p.i. We could not detect the virus on day 3 p.i. On days 5 and 6 p.i., there were 3 × 10^4 ^and 4 × 10^5 ^PFU/mL, respectively (Table 1).

**Table 1:** Infectious viral titers in brains of dengue virus-infected C57Bl/6 mice at days 3, 5 and 6 post infection

**Days after infection (n = 3)**	**p.f.u./mL**
**3**	not detected
**5**	3.10^4 ^± 10^4^
**6**	4.10^5 ^± 10^5^

### Leukocyte recruitment is increased in the pial microvasculature of non-adapted DENV-3 infected mice

**Figure 3:**
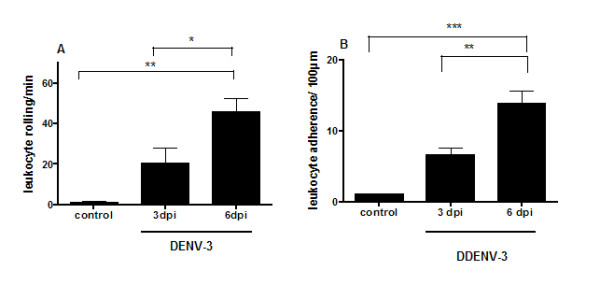
**Leukocyte-endothelium interactions visualized using intravital microscopy**. Rolling (A) and adhesion (B) of leukocytes in brain microvasculature were assessed in control mice and in mice infected with 4 × 10^3 ^PFU of DENV-3 at 3 and 6 days p.i. by the intracranial route. A higher number of rolling and adhering leukocytes was observed in the microvasculature in brain of infected mice. Data expressed as mean ± SEM of cells per minute (A) and per 100 μm (B). ***p < 0.001, **p < 0.01, *p < 0.05.

Leukocyte-endothelium interactions (rolling and adhesion) were examined in the pia-mater of control and infected mice. Meningeal vessels of control animals revealed just a limited number of rolling and adhering leukocytes. At days 3 and 6 p.i., the number of rolling and adherent leukocytes increased significantly in infected mice (Figure 3A and 3B).

### Non-adapted human DENV-3 in infected mice induces meningoencephalitis in different brain regions

**Figure 4:**
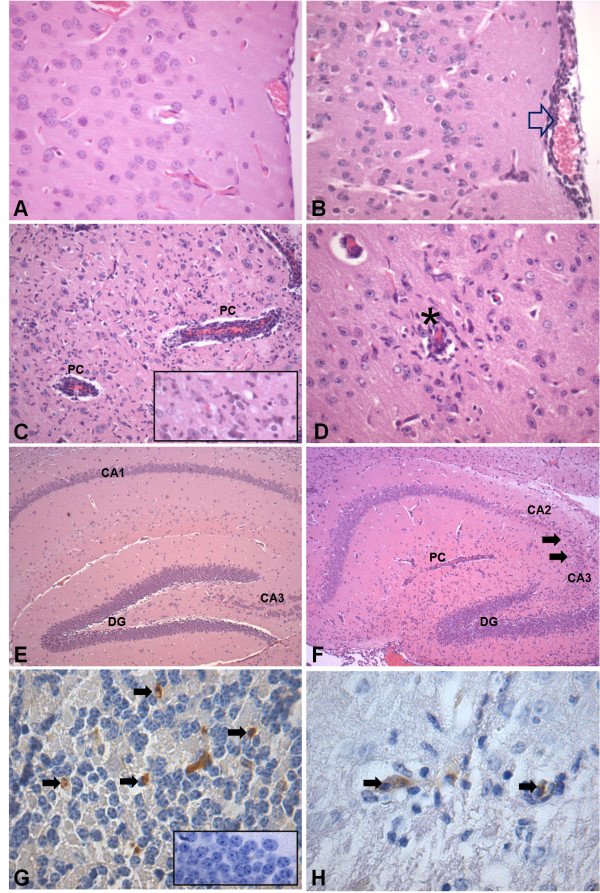
**Histopathological changes in brain of control mice (n = 4) and of mice infected with 4 × 10^3 ^PFU of DENV-3 (n = 4) by intracranial route, 6 days p.i**. Hematoxilin & eosin-stained sections from cerebrum of a control animal with normal histological appearance of cerebrum **(A) **and hippocampus **(E)**. Brain sections from a DENV-3-infected animal showing: meningoencephalitis characterized by infiltration of immune cells in the meninges (open arrow) **(B)**, perivascular cuffing (PC) **(C)**, gliosis (insert) and **(D) **vasculitis (asterisk). Note intense neuronal destruction in the pyramidal layer of the CA3 and CA2 areas of the hippocampus **(F)**. Immunohistochemical staining for anti-NS3 protein for DENV-3 showing immunoreactive cells (arrows) in the cerebellar granular layer **(G) **and in the inflammatory area of cerebrum **(H)**. The insert in G is a negative control from the cerebellum of a non-infected animal. Original magnification: A-B, C (insert), D: x400; C: x200; E-F: x100; G (insert)-H, x1000.

**Figure 5:**
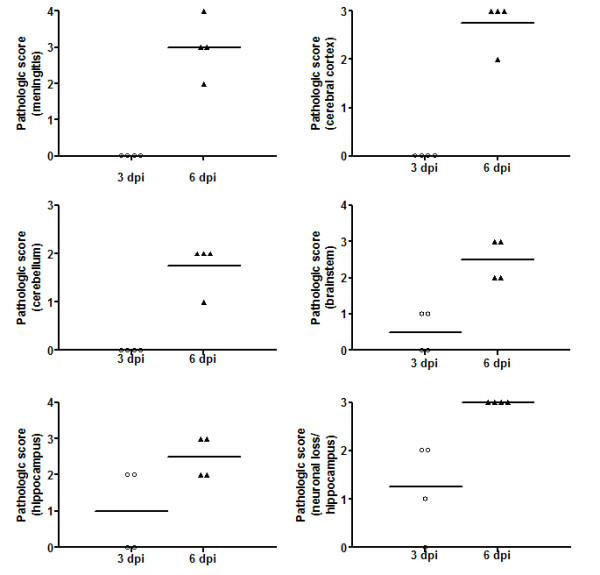
**Pathology scores for the brain after DENV-3 infection (C)**. Morphological analysis was performed on coded samples of different brain areas (cerebrum, cerebellum, hippocampus, brainstem and meninges) at 3 and 6 days p.i. These show progressive pathological changes after DENV-3 infection. Horizontal bars represent median scores.

Brains from mice that were given PBS (control) or from mice that were infected with DENV-3 were removed at days 3 and 6 p.i. Control animals did not show any histological changes (Figure 4A and 4E). None of the infected animals presented meningitis at day 3 p.i. However, mild gliosis and multifocal areas of perivascular hemorrhages were detected in the brainstem of all animals. On day 6 p.i., all animals showed diffuse meningeal infiltration of neutrophils and mononuclear cells in cerebrum and cerebellum (Figure 4B). Several perivascular cuffs of mononuclear cells, numerous rod-shaped microglial cells (Figure 4C) and vasculitis (Figure 4D) were widely distributed in the neuropil of cerebrum, hippocampus and brainstem. The hippocampus showed intense neuronal destruction of the pyramidal layer from the CA3 and CA2 areas (Figure 4F, Figure 5). NS3-positive cells were visualized throughout the parenchyma from cerebrum, brainstem and cerebellum (Figure 4G). These cells were mainly located in foci of tissue inflammation and destruction (Figure 4F).

We compared the extent of brain pathology in meninges, cortex, brainstem, hippocampus and cerebellum of mice following infection. We used a four-point scale to evaluate the effect of DENV-3 on encephalitis. This semi-quantitative analysis demonstrated progressive pathological changes in the meninges, cerebral cortex, cerebellum, hippocampus and brainstem (Figure 5).

### Cytokine and chemokine levels are increased in brain tissue from non-adapted DENV-3 infected mice

**Figure 6:**
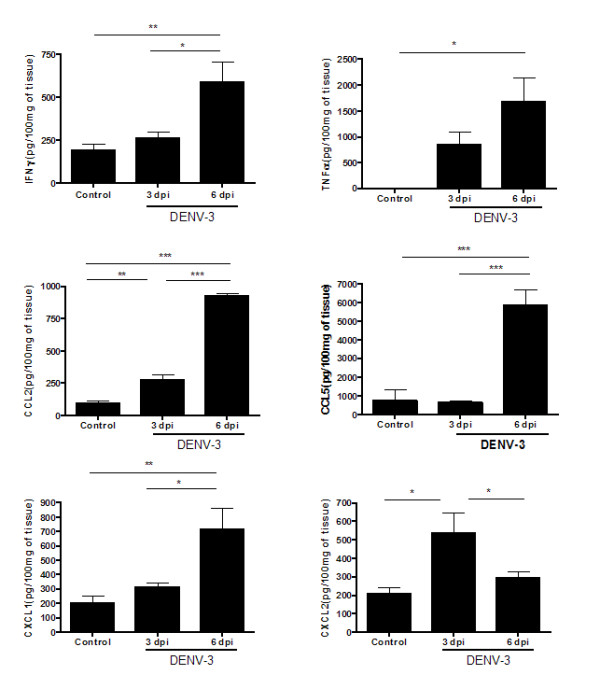
**DENV-3 infection induces cytokines (IFN-γ and TNF-α) and chemokines (CCL2, CCL5, CXCL1, CXCL2) in brain of control mice and of mice infected by the intracranial route with 4 × 10^3 ^PFU of DENV-3 (n = 9, for each time point)**. Infected mice exhibit high levels of cytokines and chemokines during infection when compared with control animals. Data expressed as mean ± SEM. ***p < 0.001, **p < 0.01, *p < 0.05.

We evaluated cerebral levels of the cytokines IFN-γ and TNF-α, and of the chemokines CCL2, CCL5, CXCL1, and CXCL2 (Figure 6). Following DENV-3 infection, early chemokine expression is marked mainly by the production of CXCL2 and CCL2, responsible for attracting neutrophils and monocytes, respectively. The levels of CCL2 were persistently elevated throughout the course of DENV-3 infection. After this initial expression, increases in the levels of CXCL1, CCL5, TNF-α, and IFN-γ occurred.

### Neutrophils are increased in the brains of non-adapted DENV-3 infected mice

**Figure 7:**
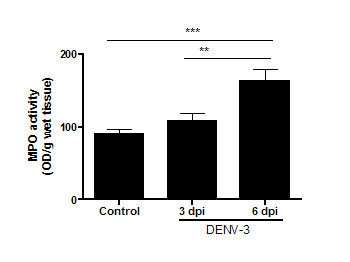
**MPO levels in cerebral tissue of control mice and mice infected by the intracranial route with 4 × 10^3 ^PFU of DENV-3**. Infected mice showed increased NAG levels on day 6, when compared with the control mice. Data represent results from groups of at least five mice, and results are expressed as mean ± SEM. ***p < 0.001; **p < 0.01.

To confirm the presence of neutrophil infiltration in brains of DENV-3 infected mice, we measured MPO activity in brain tissue from control and infected mice at days 3 and 6 p.i. Levels of MPO activity progressively increased during the course of infection. MPO activity was significantly lower in control mice when compared with the infected group (Figure 7).

### DENV-3 infection promotes increased levels of neutrophils and lymphocytes

**Figure 8:**
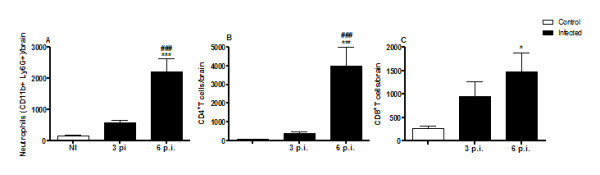
**Brain-sequestered cell numbers in C57Bl/6 mice of control mice (n = 6) and of mice infected with DENV-3**. C57Bl/6 mice were infected with 4 × 10^3 ^PFU of DENV-3 by the intracranial route, and assessed at days 3 (n = 4) and 6 p.i. (n = 4). Brain-sequestered cells were counted and then stained with specific antibodies. Flow cytometry, with assessment according to size and granularity, was performed as analysis. Numbers of neutrophils (A), and CD4^+ ^(B) and CD8^+ ^(C) T lymphocytes were evaluated in WT mice. Results are expressed as mean ± SEM, and *p < 0.05, ***p < 0.001 when compared to non-infected mice. ###p < 0.001 when compared to 3 p.i. infected mice.

Fluorescence-activated cell sorting (FACS) analysis was used to determine the specific immune cell composition in infected brains, targeting neutrophils, CD4^+ ^and CD8^+ ^T-cells (Figure 8). Control mice had fewer CNS inflammatory cells, including neutrophils, CD4^+^, and CD8^+ ^T-cells compared to infected mice. The brain of infected animals at day 6 p.i. contained significantly higher numbers of neutrophils, characterized as CD11^+ ^Ly6G^+^, compared with infected mice at day 3 p.i (Figure 8A) (p < 0.001). On day 3 post infection, the number of CD4^+ ^T-cells was similar in both groups (control versus infected) (Figure 8B). Otherwise, the number of CD8^+ ^T-cells was approximately four-fold higher among infected mice, compared with control mice at the same period (Figure 8C). Moreover, we found that the number of CD4^+ ^as well as CD8^+ ^T-cells was significantly elevated in DENV-3 mice at day 6 p.i. (p < 0.001 and p < 0.02, respectively).

## Discussion

In this study, we found that DENV-3 infected mice developed lethal encephalitis. Behavioral changes occurred at the peak of inflammatory changes in the CNS of infected animals and preceded death. To our knowledge, behavioral changes associated with leukocyte trafficking, the population of immune cells recruited into the brain, and cytokine/chemokine expression in DENV-3 encephalitis have not been previously reported.

In the present model, non-adapted DENV-3 was inoculated by an intracranial route to evaluate the CNS immune response to the infection. This route allows the study of viral-associated CNS lesions which are not seen in mice inoculated by an intraperitoneal route [20]. The observed CNS lesions were similar to those seen in human dengue encephalitis [6]. Using a similar approach, we have demonstrated that the intracranial route allows investigation of specific immune mechanisms involved in the CNS response to viral infection which are not evident when HSV-1 is inoculated in the periphery [12].

Our results also provide evidence of CNS compromise using a standardized protocol which demonstrated changes in muscle tone and strength, and motor behavior. Similar neurological deficits have been reported in patients with dengue infection [5]. We also found that these behavioral changes occurred after the increase of the inflammatory response and tissue destruction in CNS. Furthermore, the infected animals presented neurological symptoms of apathy, stereotyped behavior, and seizures, characterized by forelimb clonus with rearing and falling. At day 6 p.i. we observed greater pathologic brain scores with loss of more than 30% of neurons in the hippocampus, mainly in the CA3 region. Apoptotic cell death has been described in several flaviviral infections, such as dengue viruses, Japanese encephalitis virus and West Nile Virus (WNV). DENV-2 triggers apoptotic signaling to kill infected cells and initiate survival signaling to hold the cells in a favorable condition for longer virus progeny production. Similar pathways could also be involved in DENV-3 encephalitis [21]. DENV-2 activates the PI3K/Akt pathway as an antiapoptotic pathway to protect infected cells from early apoptotic cell death [22]. Infection with WNV in the CNS causes limbic seizures with participation of IFN-γ in the development of excitatory glutamate receptor-responsive circuits in the CNS, especially involving the N-methyl-D-aspartate (NMDA) receptor. NMDA inhibition not only abrogates limbic seizures, but also prolongs survival of infected animals. The modulation of these neurological pathways has not been evaluated here but may have important implications for patients suffering from dengue encephalitis in the clinical setting [23]. Further studies should investigate this possibility.

DENV-3-infected mice exhibited progressive meningoencephalitis characterized by infiltration of neutrophils and mononuclear cells. Before migrating to the brain parenchyma, leukocytes must roll and adhere to the brain microvasculature [24]. We assessed leukocyte rolling and adhesion in pia-mater vessels at days 3 and 6 p.i. Leukocyte recruitment was up-regulated in the microvasculature, leading to increased infiltration of inflammatory cells into brain tissue. Recruitment of these cells into the CNS may be the result of chemoattraction exerted by increased levels of cytokines and/or chemokines [10].

CXCL2, a chemokine related to preferential recruitment of neutrophils, was increased at 3 and 6 days p.i., indicating the presence of stimulus for leukocyte recruitment during the course of the infection. CXCL2 has been associated with exacerbation of brain damage via neutrophil-dependent mechanisms [25]. It is highly expressed in sera of dengue fever and dengue hemorrhagic fever patients, as well as in *in vitro *models of DENV infection [26,27]. In the lethal encephalitis induced by the JHM strain of mouse hepatitis virus (JHMV), neutrophils enter the CNS at least 24 h before other infiltrating mononuclear cells and remain the dominant inflammatory cell population throughout infection. Infection with JHMV in neutropenic animals results in increased levels of virus replication and mortality. Furthermore, neutropenia is associated with reduced infiltration of all inflammatory cells, showing that neutrophils play a key role in promoting infiltration of mononuclear inflammatory cell populations in response to CNS viral infection [28]. A paradoxical role for neutrophils has been reported in the pathogenesis of WNV [29]. Neutrophils have a biphasic role in WNV infection, serving as a reservoir for replication and dissemination in early infection and later contributing to viral clearance. Also, the immune response to dengue virus may involve cell infiltration at two moments, since neutrophils are visualized in early and late stages of brain inflammation after DENV-3 infection. Further studies are needed to dissect the role of neutrophils along the disease course.

DENV-3-infected mice presented increased brain levels of CCL2, CCL5, CXCL1, as well as of the cytokines TNF-α and IFN-γ, at 6 days p.i. The production of these proteins possibly created a more favorable milieu for cell migration and may themselves be produced by migrated cells. The early and late expression of CXCL1 and CXCL2 suggests that there is a continuous recruitment of neutrophils to the brain, which was confirmed by histopathology, MPO and FACS results. CCL5 is a member of the CC chemokine family and recruits monocytes and T cells via the chemokine receptors CCR1, CCR3, and CCR5. CCL5 has also been reported to be up-regulated during dengue infection [30]. We recently demonstrated that CCR1, CCR2 and CCR4 have minor effects in the pathogenesis of disease in a model of DENV-2 in mice inoculated by i.p. route. It appears that these receptors do not play an essential role in protection against primary infection, suggesting that the chemokine storm that follows severe primary dengue infection correlates primarily with development of disease rather than protection against severe infection [31].

We found increased levels of TNF-α in mouse brain tissue just before the onset of clinical signs of encephalitis. It is well known that higher concentrations of TNF-α correlate with severe dengue disease *in vivo *and high viral titers *in vitro *and *in vivo *[32]. Higher levels of TNF-α in infected mice have been associated with endothelial activation [33,34]. During viral encephalitis, MMPs can affect inflammatory responses by processing molecules like TNF-α, mediating transmigration of leukocytes and the development of CNS damage [13]. In DENV-3 infected mice, enhanced levels of TNF-α may reflect activation of endothelial cells, which in turn leads to an increase in the number of rolling and adhered leukocytes seen by intravital microscopy. These results are in accordance with *in vitro *studies that have demonstrated TNF-α up-regulation of polymorphonuclear cell adhesion to cerebral endothelium [35]. Increased levels of TNF-α may also be involved in the behavioral signs detected on day 6 p.i. The cytokines IL-1-β, IL-6, and TNF-α have been associated with cognitive processes such as synaptic plasticity, neurogenesis, and neuromodulation [36].

Another important cytokine up-regulated in DENV-3 infected mice was IFN-γ. IFN-γ plays a crucial role in the ability of the murine host to deal with dengue infection. High levels of IFN-γ are observed in patients with dengue and are associated with severity of the disease [37,38]. Dengue hemorrhagic fever induced by DENV-3 has been associated with higher viremia early in illness and earlier peak plasma IFN-γ levels; maximum plasma viremia levels correlate with degree of plasma leakage and thrombocytopenia [39]. Some researchers have demonstrated that IFN-deficient mice are more susceptible to dengue infection [40]. We have previously detected high levels of IFN-γ, together with IL-6 and CCL2, in brain homogenates and sera of mice infected with DENV-3 genotype I, confirming its virulence and immunogenicity in the brain [41]. The secretion of the cytokine IFN-γ by CD8^+ ^T-cells plays a role in the defense against DENV-2 [42]. We also observed a progressive increase of IFN-γ associated with high numbers of CD8^+ ^T-cells in brain tissue, showing also the importance of this cytokine during infection with DENV-3. Some experimental studies have provided evidence that glucocorticoid treatment can have beneficial effects, but this may also exacerbate the pathogenesis of viral encephalitis. The use of glucocorticoids during viral encephalitis can restrain inflammation and microglial reactivity, and decrease cerebral damage due to viral replication. However, such therapy initiated before the establishment of an appropriate acute-phase response may increase neurovirulence and CNS damage [43].

Other cytokines may also be involved in the pathogenesis of DENV-3 infection. IFN type I (α/β), which is produced by many cells, is crucial for the immediate control of initial viral replication and powerfully initiates innate and specific immune responses [44]. Studies with mice infected with DENV-2 by intravenous route have demonstrated that IFN-α/β receptor-mediated action limits initial virus replication in extraneural sites and controls subsequent viral spread into the CNS. In contrast, IFN-γ receptor-mediated responses seem to act at later stages of dengue disease by restricting viral replication in the periphery and eliminating virus from the CNS [45]. The mechanisms by which the IFN system mediates the antiviral response in mice after intracranial inoculation with DENV-3 have not yet been elucidated. Further experiments are necessary to clarify the role of IFNs during meningoencephalitis caused by DENV.

In conclusion, the present study shows that neuroinflammatory changes lead to alterations in motor behavior and muscle tone and strength in DENV-3-infected mice. The neuroinflammatory process is marked by up-regulation of the chemokines CCL2, CCL5, CXCL1, and CXCL2, and of the cytokines TNF-α and IFN-γ, which occurs in parallel with increased leukocyte rolling and adhesion in meningeal vessels and infiltration of immune cells into the brain. The inflammatory response may play a key role in the development of severe neurological manifestations in dengue disease.

## Competing interests

The authors declare that they have no competing interests.

## Authors' contributions

DCGA carried out behavioral tests, immunological assays and drafted the first version of the manuscript. MAR and MCV performed the histopathological analysis and contributed to write the manuscript. RDLC, DHR, NLQ, ASM participated in the behavioral tests, immunological assays and intravital microscopy analysis. GPF was responsible for the inoculation and quantification of the virus. VVC performed the immunohistochemistry analysis. MAC and EGK participated in the design and coordination of the study. MMT and ALT designed the study and were responsible for the interpretation of experiments and editing the manuscript. All authors have read and approved the final version of the manuscript.
